# Salvage Boron Neutron Capture Therapy for Malignant Brain Tumor Patients in Compliance with Emergency and Compassionate Use: Evaluation of 34 Cases in Taiwan

**DOI:** 10.3390/biology10040334

**Published:** 2021-04-15

**Authors:** Yi-Wei Chen, Yi-Yen Lee, Chun-Fu Lin, Po-Shen Pan, Jen-Kun Chen, Chun-Wei Wang, Shih-Ming Hsu, Yu-Cheng Kuo, Tien-Li Lan, Sanford P. C. Hsu, Muh-Lii Liang, Robert Hsin-Hung Chen, Feng-Chi Chang, Chih-Chun Wu, Shih-Chieh Lin, Hsiang-Kuang Liang, Jia-Cheng Lee, Shih-Kuan Chen, Hong-Ming Liu, Jinn-Jer Peir, Ko-Han Lin, Wen-Sheng Huang, Kuan-Hsuan Chen, Yu-Mei Kang, Shueh-Chun Liou, Chun-Chieh Wang, Ping-Ching Pai, Chih-Wei Li, Daniel Quah Song Chiek, Tai-Tong Wong, Shih-Hwa Chiou, Yee Chao, Hiroki Tanaka, Fong-In Chou, Koji Ono

**Affiliations:** 1 Faculty of Medicine, National Yang Ming Chiao Tung University, Taipei City 112304, Taiwan; chen6074@gmail.com (Y.-W.C.); yylee6@vghtpe.gov.tw (Y.-Y.L.); mmmmeeeii@gmail.com (Y.-M.K.); sjliou@vghtpe.gov.tw (S.-C.L.); 2 Department of Oncology, Taipei Veterans General Hospital, Taipei City 11217, Taiwan; yukatakids@hotmail.com (T.-L.L.); happyken176@gmail.com (J.-C.L.); skchen@vghtpe.gov.tw (S.-K.C.); ychao@vghtpe.gov.tw (Y.C.); 3 Department of Medical Imaging and Radiological Technology, Yuanpei University of Medical Technology, Hsinchu City 30015, Taiwan; 4 Department of Neurosurgery, Taipei Veterans General Hospital, Taipei City 11217, Taiwan; cf_lin@vghtpe.gov.tw (C.-F.L.); doc3379b@gmail.com (S.P.C.H.); 5 Department of Chemistry, Tamkang University, New Taipei City 251301, Taiwan; 138020@mail.tku.edu.tw; 6 Institute of Biomedical Engineering and Nanomedicine, National Health Research Institutes, Miaoli County 350401, Taiwan; jkchen@nhri.org.tw; 7 Department of Oncology, National Taiwan University Hospital, Taipei City 100229, Taiwan; elvin@pie.com.tw (C.-W.W.); tonyliang@ntuh.gov.tw (H.-K.L.); 8 Department of Biomedical Imaging and Radiological Sciences, National Yang Ming Chiao Tung University, Taipei City 112304, Taiwan; smhsu@ym.edu.tw; 9 Department of Radiotherapy, China Medical University Hospital, Taichung City 404327, Taiwan; shapico22@gmail.com; 10 Department of Neurosurgery, Mackay Memorial Hospital, Taipei City 104217, Taiwan; liang4617@hotmail.com (M.-L.L.); hhchen3@vghtpe.gov.tw (R.H.-H.C.); 11 Department of Radiology, Taipei Veterans General Hospital, Taipei City 11217, Taiwan; fcchang@vghtpe.gov.tw (F.-C.C.); ccwu6@vghtpe.gov.tw (C.-C.W.); 12 Department of Pathology and Laboratory Medicine, Taipei Veterans General Hospital, Taipei City 11217, Taiwan; diegolin@vghtpe.gov.tw; 13 Nuclear Science & Technology Development Department, National Tsing-Hua University, Hsinchu City 30013, Taiwan; hmliu@mx.nthu.edu.tw (H.-M.L.); jjpeir@mx.nthu.edu.tw (J.-J.P.); 14 Department of Nuclear Medicine, Taipei Veterans General Hospital, Taipei City 11217, Taiwan; khlin1979@gmail.com (K.-H.L.); wshuang2@vghtpe.gov.tw (W.-S.H.); 15 Department of Pharmacy, Taipei Veterans General Hospital, Taipei City 11217, Taiwan; khchen3@vghtpe.gov.tw; 16 Department of Radiation Oncology, Chang-Gung Memorial Hospital, Linkou Dist, New Taipei City 333011, Taiwan; jjwang@cgmh.org.tw (C.-C.W.); ss0122@cgmh.org.tw (P.-C.P.); 17 Delicate Clinic, Taishan Dist, New Taipei City 243081, Taiwan; kib175@gmail.com; 18 Division of Radiation Oncology, National Cancer Centre, Singapore 169610, Singapore; daniel.quah.s.c@singhealth.com.sg; 19 Department of Neurosurgery, Taipei Medical University Hospital, Taipei City 110301, Taiwan; ttwong99@gmail.com; 20 Department of Medical Research, Taipei Veterans General Hospital, Taipei City 11217, Taiwan; shchiou1130@gmail.com; 21 Institute for Integrated Radiation and Nuclear Science, Kyoto University, Osaka Prefecture 590-0494, Japan; h-tanaka@rri.kyoto-u.ac.jp; 22 Kansai BNCT Medical Center, Osaka Medical College, Takatsuki City, Osaka Prefecture 569-8686, Japan

**Keywords:** BNCT, glioblastoma, T/N ratio, T/B ratio, radioresistance

## Abstract

**Simple Summary:**

Although boron neutron capture therapy (BNCT) is a promising therapeutic approach for malignant brain tumors, the optimal BNCT parameters for patients with life-threatening, end-stage brain tumors remain unclear. The results of this study show that for life-threatening, end-stage brain tumor patients, BNCT is a promising therapeutic modality that is associated with an overall survival time of 7.25 months and no severe adverse events (grade ≥ 3). Remarkably, patients who achieved a complete response had overall survival times and cancer-specific survival times of up to 17.66 and 22.5 months, respectively. In addition, since these patients are usually physically weak and already on the verge of life-threatening conditions, reducing the BNCT dose still has good therapeutic outcomes. Statistical analysis revealed the optimal BNCT parameters and tumor characteristics, including a tumor-to-normal tissue (T/N) uptake ratio of ≥4, a tumor volume of <20 mL, a mean tumor dose of ≥25 Gy-E, MIB-1 ≤ 40, and a lower recursive partitioning analysis (RPA) class. The results of this study provide a reference for other clinicians or radiation oncologists conducting BNCT treatment for such patients.

**Abstract:**

Although boron neutron capture therapy (BNCT) is a promising treatment option for malignant brain tumors, the optimal BNCT parameters for patients with immediately life-threatening, end-stage brain tumors remain unclear. We performed BNCT on 34 patients with life-threatening, end-stage brain tumors and analyzed the relationship between survival outcomes and BNCT parameters. Before BNCT, MRI and ^18^F-BPA-PET analyses were conducted to identify the tumor location/distribution and the tumor-to-normal tissue uptake ratio (T/N ratio) of ^18^F-BPA. No severe adverse events were observed (grade ≥ 3). The objective response rate and disease control rate were 50.0% and 85.3%, respectively. The mean overall survival (OS), cancer-specific survival (CSS), and relapse-free survival (RFS) times were 7.25, 7.80, and 4.18 months, respectively. Remarkably, the mean OS, CSS, and RFS of patients who achieved a complete response were 17.66, 22.5, and 7.50 months, respectively. Kaplan–Meier analysis identified the optimal BNCT parameters and tumor characteristics of these patients, including a T/N ratio ≥ 4, tumor volume < 20 mL, mean tumor dose ≥ 25 Gy-E, MIB-1 ≤ 40, and a lower recursive partitioning analysis (RPA) class. In conclusion, for malignant brain tumor patients who have exhausted all available treatment options and who are in an immediately life-threatening condition, BNCT may be considered as a therapeutic approach to prolong survival.

## 1. Introduction

Malignant gliomas, which occur in the brain and spinal cord, are the most common primary malignant tumors found in adults worldwide. Malignant gliomas not only affect brain function, but can also be life-threatening, depending on where and how fast they grow. Among them, glioblastoma, also known as WHO grade IV astrocytoma, is the most malignant subtype of all brain tumors and has an extremely poor survival rate [1]. The current standard treatment for glioblastoma is maximal surgical resection, followed by combined radiotherapy and temozolomide (TMZ) chemotherapy. However, most patients still experience tumor progression very quickly, with a survival time of about 14 months and a 5-year survival rate of less than 5% [2]. Although bevacizumab has been approved by the Food and Drug Administration USA in 2009 for the treatment of glioblastoma in adults [3], several clinical trials have shown that bevacizumab did not improve overall survival (OS), but did improve the progression-free survival (PFS) of patients with newly diagnosed glioblastoma or progressive glioblastoma [4,5]. In addition, most glioblastoma patients receiving bevacizumab progressed rapidly, with a median time of 3–5 months [6]. Thus, it is necessary to provide another treatment strategy to control the tumor volume and prolong survival.

Boron neutron capture therapy (BNCT) has recently been considered a promising cancer treatment that exploits the neutron capture and fission reaction of the boron isotope ^10^B [7,8,9]. When tumor cells absorb nonradioactive ^10^B and are then irradiated with thermal neutrons, high linear energy transfer (LET) alpha particles (^4^He) and recoiling lithium-7 (^7^Li) are generated to damage tumor cells. Due to the short path lengths of the high energy particles, damage is limited to the tumor cells and does not affect the surrounding normal cells. BNCT is based on the preferential accumulation of ^10^B derivatives such as ^10^B-L-BPA in tumor cells relative to surrounding normal cells. The tumor-to-normal tissue uptake ratio (T/N ratio) and the tumor-to-blood uptake ratio (T/B ratio) of boron-containing agents are often used as indicators of BNCT treatment. On the other hand, in addition to the clinicopathologic features of patients, such as tumor type, tumor location, tumor size, Karnofsky performance status (KPS), and recursive partitioning analysis (RPA), other key issues affecting BNCT outcomes include the gray-equivalent radiation dose (Gy-E) and blood boron concentration during neutron irradiation [10].

In recent years, BNCT has been performed on patients with newly diagnosed glioblastoma, recurrent malignant glioma, high-grade meningioma and diffuse intrinsic pontine glioma, with mostly satisfactory survival outcomes [11,12,13,14]. However, to the best of our knowledge, few studies have focused on the effectiveness of using BNCT in patients diagnosed with terminal-stage brain cancer or patients with brain tumors who have declared various treatments to be ineffective. Thus, the present study included 34 cases of advanced terminal-stage or treatment-failure brain tumors to identify the optimal BNCT parameters and proper clinicopathologic features to understand the realistic effect of BNCT therapy on malignant brain tumors.

## 2. Methods

From March 2017 to April 2019, a total of 34 patients with malignant brain tumors were treated with BNCT. All patients were in an immediately life-threatening condition and met the criteria for Emergency and Compassionate Use (also known as Expanded Access Program) approved by the institutional review board of Taipei Veterans General Hospital (Approval number: #2020-11-002B; Project number: #TBCGHPA20200724R) and Taiwan Food and Drug Administration (TFDA), Ministry of Health and Welfare R. O. C. All of these eligible patients had severe and life-threatening brain tumors, and the doctor confirmed that there was no other comparable or satisfactory treatment for the patient. BNCT was administrated as a compassionate treatment with written informed consent from patients or authorization from their parents. Carson recursive partitioning analysis (RPA) classification was used to classify the BNCT cases. The grade or proliferative activity of these brain tumors was determined by the MIB-1 proliferation index, which indicates the percentage of MIB-1-positive tumor nuclei among the tumor nuclei counted.

Magnetic resonance imaging (MRI) was used to identify and confirm the presence of brain lesions in patients, and ^18^F-BPA-positron emission tomography (PET) was used to determine the ^18^F-^10^B-L-BPA distribution and boron concentration in tumors. The T/N and T/B ratios of ^18^F-^10^B-L-BPA were determined and recorded. We took the contralateral lobe of the brain as normal tissue and used this in the T/N ratio calculation. The heart ventricle was used as a blood pool to determine the T/B ratio. The mean standard uptake value (SUVmean) was used for the tumor lesion. Herein, Gy-E was used to indicate the biologically equivalent dose administrated to patients during BNCT. Gy-E values were calculated by multiplying the physical dose given by the relative biological effectiveness (RBE). The compound biological effectiveness (CBE) was used to assess the boron dose derived from effects of the ^10^B(n, α)^7^Li reaction employing ^10^B-L-BPA as the boron carrier. The following values were used in this study: RBE-thermal neutron = 3.2, RBEγ = 1.0, CBE = 3.8 for the tumor, CBE = 2.5 for normal skin, CBE = 1.35 for the nerves, brain max dose < 10 Gy-E, and brain mean dose < 2 Gy-E.

The BNCT protocol used in this study was modified based on the regimen developed by Kyoto University in Japan [15,16]. The total infused dose was 450 mg/kg body weight. The infusion formula was full infusion for the first two hours (180 mg/kg) and then infusion at half of the initial infusion rate (90 mg/kg) during neutron irradiation to maintain a constant boron concentration in the blood [15,17]. The epithermal neutron source for BNCT was the Tsing-Hua Open-Pool Reactor (THOR) at National Tsing-Hua University [18]. The blood boron concentration and minimum, maximum, and mean tumor doses required for the gross tumor volume (GTV) were estimated and recorded. In this study, the mean tumor dose required for the gross tumor volume (GTV) was estimated to be 20–40 Gy-E. Bevacizumab was administered at a dose of 10 mg/kg immediately after BNCT and at two-week intervals until disease progression, severe treatment-related toxicity (grade ≥ 3), or mortality of the patient.

The response assessment in neuro-oncology criteria (RANO) were used to assess the tumor response after BNCT by two senior neuroradiologists. All patients were followed-up by MRI examination to assess their brain tumors. MRI examination was performed 1 month after BNCT, and follow-up MRIs were performed every 3 months thereafter. In addition, adverse events were recorded according to the Common Terminology Criteria for Adverse Events (CTCAE) version 5 [19]. Overall survival (OS) was defined as the time elapsed from the day of intervention to the date of death or the last follow-up, with no restriction on the cause of death. Cancer-specific survival (CSS) was defined as the time elapsed from the day of intervention to the date of death due to brain tumor rather than other causes. Relapse-free survival (RFS) was defined as the time interval between the day of intervention and the date of relapse/recurrence (regional or distant metastases) or death (all causes), whichever occurred first.

## 3. Results

### 3.1. Patient Characteristics and BNCT Parameters

A total of 34 patients with malignant brain tumors were treated with BNCT. The demographic characteristics and BNCT parameters are presented in Table 1. The mean age of patients was 37.5 ± 20.5 years, and the male to female ratio was 1:1.27. Glioblastoma (44.1%) and astrocytoma (32.4%) were the major tumor types. Most tumors were located in the non-frontal lobe, and the average tumor size was 58.19 ± 61.14 mL. Microscopic analysis revealed that the MIB-1 index of the tumors was 37.35 ± 16.04%. The median KPS score was 70. The RPA class distribution of these patients before BNCT treatment at the initial diagnosis was as follows: Class I (15.2%), Class II (18.2%), Class III (0.0%), Class IV (18.2%), Class V (27.3%), and Class VI (21.2%). Most patients were classified in RPA classes IV–VI (66.7%). Before receiving BNCT treatment, the comorbidities of these patients included epilepsy (34.3%), diabetes mellitus (11.4%), sinus bradycardia (5.7%), panic (5.7%), hyperlipidemia (5.7%), arrhythmia (5.7), mismatch repair syndromes (5.7%), asthma (11.4%), and abducent palsy (2.9%). During BNCT treatment, the average T/N ratio was 2.93 ± 0.98, while the average T/B ratio was 2.67 ± 0.94. The boron concentration in the blood during epithermal neutron irradiation was 26.75 ± 4.99 ppm, and the mean tumor dose was 17.44 ± 7.50 Gy-E (mean physical dose = 5.75 ± 2.29 Gy). Most patients (82.4%) received bevacizumab after BNCT treatment.

### 3.2. The Association between Tumor Response and Survival Outcomes

The tumor response of patients after three months of BNCT treatment was assessed by cranial MRI. The proportions of patients who achieved a complete response (CR), partial response (PR), stable disease (SD), and progressive disease (PD) were 17.6%, 32.4%, 35.3%, and 14.7%, respectively (Table 2). The objective response rate (ORR) was 50%, and the disease control rate (DCR) was 85.3%.

The association between tumor responses and the median OS, CSS, and RFS after BNCT treatment are shown in Table 3. The median OS times for patients with CR, PR, SD, and PD were 17.43, 15.47, 6.00, and 4.83 months, respectively. The 6- and 12-month OS rates for CR patients were 100% and 64%, respectively. The 6- and 12-month OS rates for PR patients were 73% and 62%, respectively. However, patients with SD and PD had very low OS rates. Similar trends were observed for the CSS and RFS.

### 3.3. Association between BNCT Parameters and Survival Outcomes

**Figure 1:**
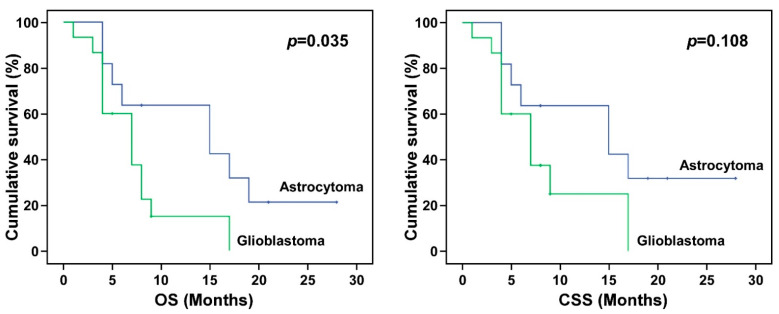
Kaplan–Meier curves for different tumor types. Kaplan–Meier survival curves for overall survival (OS) and cancer-specific survival (CSS) of patients with astrocytoma and glioblastoma. BNCT treatment showed a better OS in patients with astrocytoma than in those with glioblastoma.

**Figure 2:**
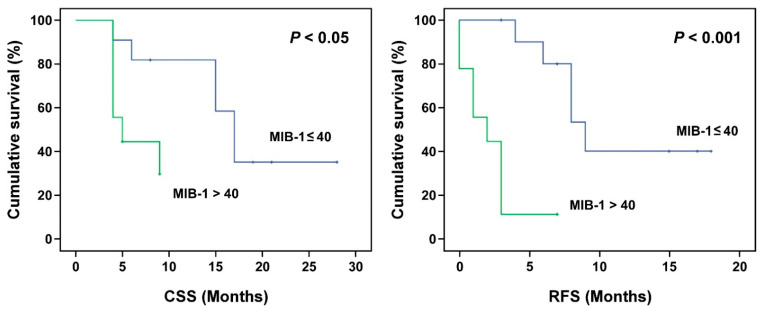
Kaplan–Meier survival curve of tumors with MIB-1 ≤ 40 and MIB-1 > 40. A MIB-1 labelling index higher than 40% predicted poor cancer-specific survival (CSS) and poor relapse-free survival (RFS).

**Figure 3:**
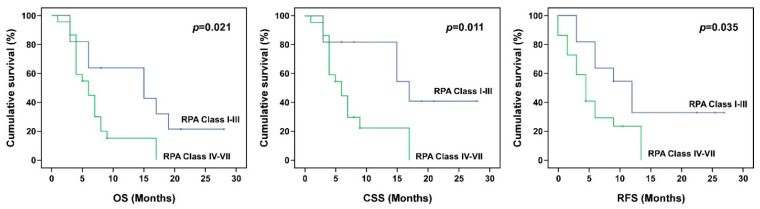
Kaplan–Meier survival curve of tumors from RPA classes I–III and RPA classes IV–VII. A higher RPA class predicted poor overall survival (OS), cancer-specific survival (CSS), and relapse-free survival (RFS).

**Figure 4:**
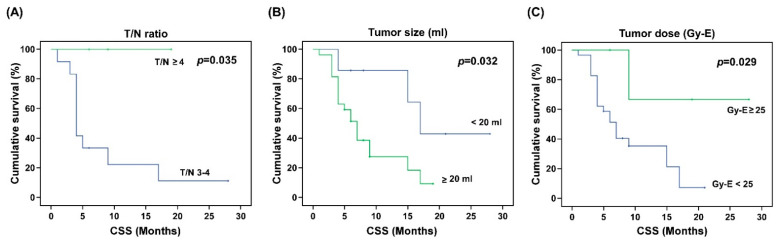
Kaplan–Meier cancer-specific survival curves of patients treated with BNCT according to the T/N ratio, tumor volume, and tumor size. (**A**) The T/N ratio denotes the tumor-to-normal tissue uptake ratio of 18F-L-BPA. Patients with a T/N ratio ≥ 4 had better CSS values than patients with a T/N ratio < 4. (**B**) Patients with a tumor volume < 20 mL or (**C**) a receiving mean tumor dose ≥ 25 Gy-E had better CSS values after BNCT treatment. *p* values are marked in the upper right corner, and *p* values of less than 0.05 were regarded as statistically significant. CSS denotes cancer specific survival.

The impacts of gender, tumor type, tumor location, MIB-1, RPA class, KPS, T/N ratio, T/B ratio, tumor volume, blood boron concentration, tumor dose, and tumor response on OS, CSS, and RFS were analyzed (Table 4). The results show that gender, tumor location, KPS, and T/B ratio had no statistically significant impacts on the OS, CSS, or RFS. Tumor type was significantly associated with the final OS (*p* = 0.003) and CSS (*p* = 0.005), but not the RFS (*p* = 0.245). The Kaplan–Meier survival analysis of tumor types further revealed that after BNCT treatment, glioblastoma patients had worse OS and CSS times than astrocytoma patients (Figure 1). Patients with high MIB-1 (>40%) had significantly worse median CSS and RFS than patients with low MIB-1 (*p* < 0.05; 6.34 vs. 13.03 months for CSS, and 0.89 vs. 7.82 months for RFS). The OS, CSS, and RFS for patients with low-risk RPA (Class I-III) and high-risk RPA (Class IV-VI) were also analyzed. Compared with patients with low-risk RPA, patients with high-risk RPA had significantly worse OS (7.04 vs. 14.58), CSS (7.74 vs. 18.41), and RFS (3.88 vs. 9.27). Kaplan–Meier survival curves for tumors with MIB-1 > 40 and MIB-1 ≤ 40 and for patients with low-risk RPA and high-risk RPA are shown in Figure 2 and Figure 3, respectively. Although the T/B ratio was not associated with survival outcomes, patients with a T/N ratio ≥ 4 had better survival outcomes than patients with a T/N ratio < 4 (Figure 4A). A smaller tumor size and high tumor dose were positively associated with the survival response following BNCT-based therapy. The receiver operating characteristic (ROC) curve was used to identify an optimal diagnostic cut-off point. The results show that patients with a tumor volume < 20 mL (Figure 4B) or receiving a mean tumor dose ≥ 25 Gy-E had better CSS (Figure 4C). The mean CSS values of patients with tumor volumes of <20 mL and ≥20 mL were 12.54 and 8.45 months, respectively (*p* = 0.032). The mean CSS values of patients receiving <25 Gy-E and ≥25 Gy-E were 9.11 and 21.67 months, respectively (*p* = 0.029). For these patients, who had immediately life-threatening conditions and complied with the Emergency and Compassionate Use standards, it is worth noting that receiving BNCT with a tumor dose ≥25 Gy-E greatly extended life by 21.67 months.

The survival rates at the 6- and 12-month time points with respect to the T/N ratio were further analyzed. As shown in Table 5, patients with T/N ≥ 4 had better 6- and 12-month survival outcomes than those with T/N < 3 or T/N 3–4. It is worth noting that patients with a T/N ratio of 3–4 had the worst OS, CSS, and RFS rates, even worse than those with a T/N ratio of < 3.

### 3.4. Adverse Reactions

The most common adverse reaction in patients was increased intracranial pressure, followed by symptoms of headache, dizziness, nausea, and vomiting. Moreover, no patients experienced severe increased intracranial pressure or a loss of consciousness. All patients experienced hair loss after BNCT treatment (Grade I in CTCAE 5.0). Except for 2 cases (5.8%), they had symptoms of somnolence after 3 months of BNCT treatment. There were no other adverse reactions, such as normal brain tissue necrosis or neurocognitive impairment.

### 3.5. Representative Case

**Figure 5:**
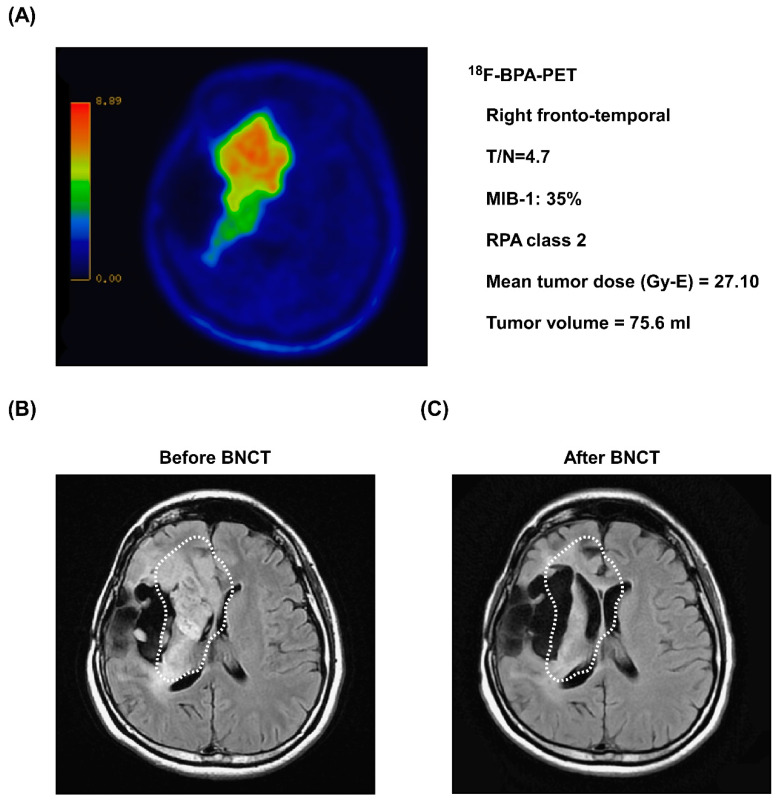
Representative images of tumor shrinkage after BNCT treatment. (**A**) Before BNCT treatment, ^18^F-BPA-PET revealed the location of strong tumor activity in the right fronto-temporal lobe. (**B**) MRI imaging of the patient before BNCT treatment. An anaplastic astrocytoma was observed in the right temporal lobe (white dashed circles). (**C**) Three months after BNCT treatment, a follow-up MRI image of the patient showed a significant reduction in tumor volume (white dashed circles).

A 35-year-old female patient was first diagnosed with anaplastic astrocytoma in the right fronto-temporal region after radical craniotomy in May 2013. After surgery, she also underwent chemoradiotherapy. However, tumor relapse with malignant transformation to glioblastoma was noted after salvage craniotomy in July 2015. The next year, the tumors relapsed rapidly, and the patient was in an immediately life-threatening condition, complying with the Emergency and Compassionate Use standards. A ^18^F-BPA-PET scan showed strong tumor activity with a high T/N ratio of 4.7 (Figure 5A). MRI imaging showed a large tumor volume (75.6 mL) in the right temporal lobe (Figure 5B). The RPA class, KPS, and MIB-1 of the patient were 2, 80, and 35%, respectively. During BNCT, the blood boron concentration and mean tumor dose were 24.7 ppm and 27.1 Gy-E, respectively. The patient received bevacizumab after BNCT. One month after BNCT, MRI showed a significant reduction in tumor volume (Figure 5C). The patient achieved a PR more than 3 months after BNCT without tumor relapse and severe adverse events (grade ≥ 3). She died outside the hospital 538 days (17.9 months) after BNCT, not because of disease progression.

## 4. Discussion

Recently, the use of BNCT has received extensive attention because of its association with highly selective tumor cell killing without causing significant damage to surrounding normal brain tissue. In addition to BNCT-based case reports, several small-scale preliminary clinical studies have demonstrated that BNCT prolongs survival outcomes with an acceptable incidence rate of adverse events in recurrent malignant gliomas [15,20] and newly diagnosed glioblastoma [21,22,23,24]. In this study, we specifically focused on the use of BNCT for brain tumor patients with immediately life-threatening disease and no other treatment options (comply with the Emergency and Compassionate Use standards). Remarkably, BNCT is effective for patients with terminal-stage malignant brain tumors. Responses, including CRs, were obtained even when a significantly restricted irradiation dose was used, and the survival rates of these patients were unexpectedly greatly extended. BNCT was shown to be a beneficial therapeutic modality for these brain tumor patients, with 50% ORR and 85.3% DCR. The 6-month OS for CR and PR patients was 100% and 73%, respectively. Remarkably, no BPA-related toxicity or serious adverse events were observed. On the other hand, we further identified the optimal parameters/clinicopathologic features for BNCT treatment in terminal-stage patients. We found that better survival outcomes were associated with a T/N ratio ≥ 4, tumor volume < 20 mL, mean tumor dose ≥ 25 Gy-E, MIB-1 ≤ 40, and lower RPA class. In addition, for BNCT-treated patients who met the standards for Emergency and Compassionate Use, those with astrocytoma had the highest survival rate, followed by those with glioblastoma.

In the BNL and Harvard-MIT clinical studies, the tolerance of the normal human brain to BNCT was found to be 6.2 Gy (w) and 14.1 Gy (w) for the whole brain and peak brain, respectively [25]. Higher doses were associated with the incidence of somnolence. In a study by Shinji KAWABATA et al., the minimal and maximal tumor BNCT doses for newly diagnosed glioblastoma cases were found to be 26.25 and 56.32 Gy-E, respectively [22]. In another study by Tetsuya Yamamoto et al., the average and maximum BNCT doses for newly diagnosed glioblastoma cases were found to be 5.1 ± 2.2 and 29.4 ± 6.0 Gy-E, respectively [24]. Most of these patients experienced erythema, low-grade fever, vasocontraction, and itching. Some patients suffered from post-epileptic brain swelling and transient orbital swelling accompanied by double vision. However, in this study, all patients complied with the Emergency and Compassionate Use standards for patients who have exhausted all available treatment options and have uncontrolled tumor development. These patients are usually physically weak, drowsy, and fatigued, but they still want to know if there are other ways to control tumor development and prolong survival. Therefore, the tumor dose used in this study was only one third to one half of that used in the Shinji KAWABATA study [22] (minimal dose 8.51 vs. 26.25; and maximal dose 25.09 vs. 56.32 Gy-E). It is worth noting that these end-stage brain tumor patients still had an average OS of 7.25 months with no severe adverse events (grade ≥ 3). Remarkably, patients who achieved a CR had OS and CSS rates of up to 17.66 and 22.5 months, respectively. Based on this study, lower BNCT doses are recommended for patients who meet the Emergency and Compassionate Use standards.

The BNCT physical dose standard (P1998) specifies a maximum GTV dose of 15 Gy, while the 2001 dose escalation plan (P2001) prescribed a minimal clinical target volume (CTV) and GTV dose of 18 Gy and 15 Gy, respectively [26]. Although some BNCT neurosurgeons prefer to use the physical dose of Gy, we herein recommend that the biologically equivalent X-ray dose concept of Gy-E is used so that the total BNCT radiation is accounted for, in which the RBE and CBE factors are carefully calculated [27]. Compared with the high-energy X-rays used in conventional X-ray radiotherapy (XRT), thermal neutrons have a lower biological effect in tissues. Most BNCT researchers believe that the LET of both helium and lithium nuclei emitted from the boron neutron capture reaction is very high and that the physical doses are equal. However, Ono et al. suggested that the effect of BPA-BNCT is determined by the nucleo-cytoplasmic ratio specific to tumor cells, and proposed the use of an absolute biological effectiveness (ABE) dose instead of a CBE dose [28]. Future research should consider the inclusion of ABE for BNCT application. On the other hand, patients who receive BNCT treatment have previously undergone repeated surgeries and conventional XRT, and thus radiation oncologists may want to estimate subsequent BNCT doses by combining the calculations of Gy-E and BNCT. However, even though BNCT combined with subsequent XRT showed promising outcomes, the equivalent dose of XRT and BNCT cannot be simply added. A study by Shinji KAWABATA et al. [22] showed that compared with receiving BNCT alone, patients with newly diagnosed glioblastoma who received BNCT followed by XRT with 20–30 Gy showed better survival outcomes (23.5 vs. 14.1 months). Similarly, Tetsuya Yamamoto et al. [24] reported that patients with newly diagnosed glioblastoma who received BNCT followed by a total X-ray dose of 30 Gy in 15 fractions or 30.6 Gy in 17 fractions showed better survival outcomes than patients who received BNCT only (25.7 vs. 11.9 months). Since most patients who meet the Emergency and Compassionate Use standards have a poor physical condition, the possibility of combining BNCT and XRT therapy needs further consideration and evaluation. In this study, the mean tumor dose for GTV was estimated to be 20–40 Gy-E.

During the initial establishment of clinical treatment with BNCT, several patients quickly developed increased intracranial pressure and died following treatment [26]. Due to the poor physical conditions of our patients, the therapeutic dose used in this study was lower than that reported in other BNCT studies. Therefore, adverse events such as increased intracranial pressure and loss of consciousness were not observed in all treatment cases. It was reported that when the tumor volume exceeds 60 cm^3^, an increased average brain dose and irradiation range may increase the incidence of adverse events. An average brain dose of 6.2 Gy-E has been reported to be associated with a 50% incidence of somnolence [24]. In this study, only two patients reported somnolence after BNCT treatment. Patients showed prolonged night-time sleep and decreased activity. However, the tumor volumes of the two cases were 5.8 and 2.3 mL, and the mean tumor doses were 35.01 and 12.46 Gy-E, respectively. In addition, brain necrosis was not observed after MRI examination, suggesting that the development of somnolence in the two cases may have been the result of normal brain tolerance in response to BNCT treatment [25]. In this study, no pseudoprogression was observed in any of the 34 cases, which may have been due to the use of bevacizumab in most patients immediately after BNCT.

Until now, the epithermal neutron source for most BNCT treatments has been a nuclear reactor. In addition to the intrinsic risk of the nuclear reactor, it is impossible to establish a reactor-based BNCT or perform subsequent treatment in a hospital. However, the miniaturized accelerator system is currently the most viable solution [29,30]. Many countries have established different types of accelerators for clinical BNCT treatment, such as cyclotron-, linac-, and electrostatic-based accelerators, most of which are located in Japan. In addition to having lower radiation hazards and miniaturization, the softer and lower-energy neutrons generated by accelerators can provide a better epithermal neutron spectrum for deep tumors [31]. Therefore, for countries or regions that currently rely on a nuclear reactor, there is an urgent need to plan and develop accelerators as alternative neuron sources as soon as possible.

**Figure 6:**
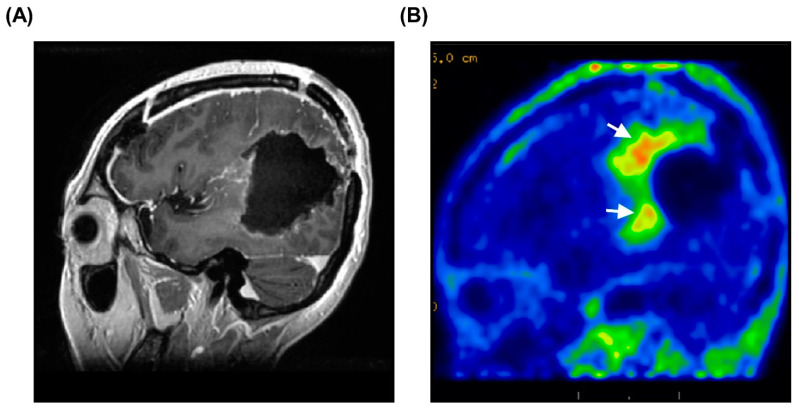
Invisible tumor activity revealed by 18F-BPA-PET. (**A**) MRI of patient after surgical resection of brain tumors. Postoperative MRI did not identify overt lesions after surgery. (**B**) Postoperative 18F-BPA-PET revealed strong tumor activity in the surgical margin. The white arrows indicate the infiltrative tumor lesions.

For successful BNCT treatment, a large accumulation of L-BPA is essential. Regarding the in vivo imaging of L-BPA accumulation, PET is an alternative technique that can be used to monitor the tumor uptake of ^18^F-BPA in patients. In other words, before performing BNCT treatment, the use of ^18^F-BPA-PET is helpful to predict the distribution and accumulation of ^18^F-BPA and screen suitable patients for BNCT [32,33,34]. In addition, ^18^F-BPA-PET can also be used to evaluate the therapeutic effect of BNCT. Figure 6A shows an MRI of a patient after complete surgical removal of the tumor. However, ^18^F-BPA-PET clearly indicated that there was still strong tumor activity at the surgical bed (Figure 6B). This indicates that surgical resection cannot completely remove infiltrative brain tumors; therefore, subsequent intensive treatment, such as BNCT, is required. On the other hand, ^18^F-BPA-PET can provide crucial information about the concentration of L-BPA in tumors and surrounding normal tissues. To ensure that the tumors are treated with the maximum radiation dose and minimal background radiation, a high T/N ratio is preferable. However, these parameters also need to be adjusted according to the current situation of the patients. In this study, we found that patients with a T/N ratio of ≥4 showed the best therapeutic outcomes. Therefore, patients who meet the conditions for an extended access plan and with T/N ≥ 4 should be listed as the first priority choice for BNCT treatment. In this study, only the association with the T/B ratio did not reach statistical significance, which may be due to the small number of patients in the study. The correlation between the T/N ratio and T/B ratio is important and should be carefully analyzed in the future.

Bevacizumab, a humanized monoclonal antibody, was first approved by the Food and Drug Administration (FDA) in USA for treating glioblastoma in 2019 [3]. Although current clinical trials showed that the survival benefit of bevacizumab for glioblastoma patients remains controversial [35,36,37], some studies demonstrated that bevacizumab can reduce radiation necrosis of patients with malignant brain tumors [38,39]. In addition, studies by Miyatake et al. [40,41] demonstrated that bevacizumab is effective in controlling radiation necrosis and symptomatic pseudoprogression encountered after BNCT treatment and may prolong the survival of recurrent malignant glioma patients. In this study, although not all patients (five patients who received BNCT only and one record missing) received BNCT combined with bevacizumab posttreatment, no radiation necrosis and pseudoprogression of the tumors was observed in all patients. Interestingly, despite the small sample size, we still found that patients received BNCT combined with bevacizumab posttreatment (*n* = 28) had better survival outcome than patients received only BNCT (*n* = 5). Compared with patients that received BNCT only, patients that received BNCT combined with bevacizumab posttreatment had significantly better OS (5.60 vs. 10.76 months, *p* = 0.041) and CSS (5.60 vs. 12.96, *p* = 0.014). However, there is no significant difference on RFS between groups (3.2 vs. 6.91, *p* = 0.188). Kaplan–Meier survival curves for patients that received BNCT only or BNCT combined with bevacizumab were shown in Supplementary Information. Although the current limited samples sizes make it difficult to consider it as biologically meaningful, our results can still support Miyatake’s results [40,41] that BNCT combined with bevacizumab may prolong the survival of patients with malignant brain tumors.

This study has two limitations. Since this study focused on patients who met the conditions for Emergency and Compassionate Use, a small population size and relatively short lifespan of the patients were limitations. The current results can only conclude that BNCT treatment is associated with a better OS in patients with astrocytoma than in those with glioblastoma. The effect of BNCT treatment on patients with other brain tumor subtypes, such as oligoastrocytoma, oligodendroglioma, meningioma, medulloblastoma, or craniopharyngioma, remain unclear. Due to the short lifespan of these patients, it is difficult to conduct a longitudinal study to investigate the long-term effects of BNCT on patients who meet the criteria for Emergency and Compassionate Use. Therefore, future studies should have prospective, rather than cross-sectional, designs and should recruit more patients to reduce the limitations associated with the study.

## 5. Conclusions

For brain tumor patients who have exhausted all available treatment options and meet the criteria for Emergency and Compassionate Use, BNCT can be considered as a therapeutic approach and might prolong the survival times of these patients. A reduced dose of BNCT for patients who meet the criteria for Emergency and Compassionate Use still has good therapeutic outcomes. BNCT showed greater efficacy in patients with astrocytoma and glioblastoma. In addition, BNCT is recommended for use in patients with a T/N ratio ≥ 4, tumor volume < 20 mL, mean tumor dose ≥ 25 Gy-E, MIB-1 ≤ 40, and lower RPA class.

## Figures and Tables

**Table 1:** Demographics of the patients before and during boron neutron capture therapy (BNCT) treatment (N = 34).

Characteristics		
Age (years)	37.50 ± 20.51	
Gender		
Female	19	(55.9%)
Male	15	(44.1%)
Tumor type		
Glioblastoma	15	(44.1%)
Astrocytoma	11	(32.4%)
Oligoastrocytoma	2	(5.9%)
Medulloblastoma	2	(5.9%)
Brainstem glioma	4	(11.7%)
Tumor location		
Frontal	7	(20.6%)
Non-Frontal in cerebrum	21	(61.8%)
Posterior fossa	6	(17.7%)
Tumor volume (mL)	58.19 ± 61.14	
MIB-1 (%)	37.35 ± 16.04	
KPS	70	(60, 80)
RPA class		
I	5	(15.2%)
II	6	(18.2%)
III	0	(0.00%)
IV	6	(18.2%)
V	9	(27.3%)
VI	7	(21.2%)
Comorbidity		
Epilepsy	12	(34.3%)
Diabetes mellitus	4	(11.4%)
Sinus bradycardia	2	(5.7%)
Panic	2	(5.7%)
Hyperlipidemia	2	(5.7%)
Arrhythmia	2	(5.7%)
Mismatch repair syndrome	2	(5.7%)
Asthma	4	(11.4%)
Abducent palsy	1	(2.9%)
T/N ratio	2.93 ± 0.98	
T/B ratio	2.67 ± 0.94	
Blood boron concentration during neutron irradiation (ppm)	26.75 ± 4.99	
Mean tumor dose (Gy-E)	17.44 ± 7.50	
Bevacizumab treatment after BNCT		
Yes	28	(82.4%)
No	5	(14.7%)

Continuous variables with normal distribution are shown as the mean ± standard deviation (SD) or median with interquartile range; categorical variables are shown as the count and percentage. The tumor volume of the patient was calculated by analyzing CT images using treatment planning software. One patient’s bevacizumab record and one patient’s RPA record were missing. Abbreviations: RT, radiotherapy; KPS, Karnofsky performance status; T/N ratio, tumor-to-normal tissue uptake ratio of the ^18^F-^10^B-L-BPA; T/B ratio, tumor-to-heart blood uptake ratio of the ^18^F-^10^B-L-BPA; Gy-E, Gray-Equivalent.

**Table 2:** BNCT treatment response (N = 34).

Response	N (%)	95% CI
CR	6 (17.6%)	0.05–0.30
PR	11 (32.4%)	0.17–0.48
SD	12 (35.3%)	0.19–0.51
PD	5 (14.7%)	0.03–0.27
ORR	50.0%	0.33–0.66
DCR	85.3%	0.73–0.97

CR, complete response; PR, partial response; SD, stable disease; PD, progressive disease; NA, not available; ORR, objective response rate (ORR = CR + PR); DCR, disease control rate (DCR = CR + PR + SD).

**Table 3:** The 6- and 12-month OS, CSS, and RFS in patients with CR, PR, SD, and PD.

	OS			CSS			RFS		
	Median Survival (Months)	6 Months	12 Months	Median Survival (Months)	6 Months	12 Months	Median Survival (Months)	6 Months	12 Months
All cases	7.25	52%	29%	7.80	58%	38%	4.18	32%	16%
CR	17.43	100%	64%	28.00	100%	50%	9.23	65%	0%
PR	15.47	73%	62%	15.47	73%	62%	8.21	55%	33%
SD	6.00	14%	0%	6.00	33%	0%	3.00	0%	0%
PD	4.83	0%	0%	4.83	40%	0%	1.50	0%	0%
ORR	15.65	82%	61%	17.11	82%	73%	8.44	59%	29%
DCR	8.10	62%	35%	9.07	62%	41%	4.57	39%	19%

OS, overall survival; CSS, cancer-specific survival; RFS, relapse-free survival; CR, complete response; PR, partial response; SD, stable disease; PD, progressive disease; NA, not available; ORR, objective response rate (ORR = CR + PR); DCR, disease control rate (DCR = CR + PR + SD).

**Table 4:** Overall survival, cancer specific survival, and relapse-free survival.

	OS			CSS			RFS		
	Mean(Month)	95% CI	*p*-Value	Mean(Month)	95% CI	*p*-Value	Mean(Month)	95% CI	*p*-Value
**Gender**									
Male	9.67	4.87, 14.46	0.758	11.32	5.56, 17.08	0.590	7.09	3.40, 10.78	0.930
Female	9.90	7.23, 12.58		10.78	8.01, 13.54		5.72	3.16 8.27	
**Tumor type**									
Astrocytoma	14.67	9.40, 19.94	**0.003**	12.91	8.15, 17.67	**0.005**	9.00	4.93, 13.07	0.245
Glioblastoma	7.34	4.84 9.83		6.47	4.56, 8.37		4.00	2.06, 5.94	
Oligoastrocytoma	-	-		-	-		2.00	2.00, 2.00	
Medulloblastoma	-	-		-	-		4.00	4.00, 4.00	
Brainstem glioma	6.75	3.76, 9.74		3.75	3.94, 9.56		4.50	2.86, 6.14	
**Tumor location**									
Frontal	11.29	5.64, 16.93	0.671	13.86	7.74, 19.97	0.440	8.71	2.67, 14.76	0.225
non-Frontal in cerebrum	9.08	5.70, 12.46		10.13	6.16, 14.10		4.00	2.65, 5.35	
Posterior fossa	9.34	4.90, 13.76		10.61	5.74, 15.49		7.40	3.82, 10.98	
**MIB-1 (%)**									
≤40	15.00	10.42, 19.59	0.086	18.21	13.03, 23.38	**0.022**	11.53	7.82, 15.25	**0.000**
>40	6.33	4.61, 8.05		6.34	4.61, 8.05		2.22	0.89, 3.56	
**RPA class**									
1–3	14.58	9.22, 19.93	**0.021**	18.41	12.50, 24.32	**0.011**	9.27	5.37, 13.17	**0.035**
4–7	7.04	5.03, 9.05		7.74	5.34, 10.14		3.88	2.46, 5.29	
**KPS**									
<70	8.75	3.81, 13.69	0.604	8.75	3.81, 13.69	0.182	4.85	1.56, 8.14	0.487
≥70	9.81	7.27, 12.34		11.30	8.57 14.02		6.19	3.69, 8.69	
**T/N ratio**									
<4	9.21	6.49, 11.92	0.283	8.23	6.05, 10.42	**0.050**	5.71	3.50, 7.92	0.507
≥4	14.67	4.86, 24.47		-	-		8.00	0.78, 15.22	
**T/B ratio**									
<4	9.77	6.94, 12.61	0.921	10.77	7.55, 13.99	0.359	5.50	3.38, 7.62	0.183
≥4	9.33	0.00, 18.96		13.67	5.13, 22.20		10.50	1.49, 19.51	
**Tumor size (mL)**									
<20	15.14	8.33, 21.96	0.056	19.43	12.54, 26.32	**0.032**	9.95	4.80, 15.11	0.091
≥20	7.92	5.78, 10.05		8.45	6.16, 10.74		4.79	2.76, 6.81	
**Mean tumor dose (Gy-E)**									
<25	8.52	6.34, 10.70	0.215	9.11	6.75, 11.47	**0.029**	5.84	3.47, 8.22	0.721
≥25	13.6	6.03, 21.17		21.67	11.53, 31.80		6.60	1.91 11.29	
**Blood boron concentration (ppm)**									
<30	10.23	7.12, 13.34	0.631	12.70	8.75, 16.66	0.305	7.13	4.50 9.75	**0.008**
≥30	6.57	4.54, 8.60		6.57	4.54, 8.60		2.14	1.24, 3.04	
**Post BNCT response**									
CR	17.66	10.20, 25.13	**0.000**	22.50	14.88, 30.12	**0.000**	7.50	5.26, 9.75	**0.009**
PR	12.78	8.90, 16.66		13.03	8.98, 17.09		8.55	4.29, 12.80	
SD	5.08	3.77, 6.39		5.08	3.77, 6.39		3.03	2.01 4.05	
PD	4.80	3.84, 5.76		4.80	3.941, 5.66		1.80	0.00, 3.60	

Bold indicates a statistically significant difference with a *p*-value less than 0.05.

**Table 5:** Post-BNCT survival rates at the 6- and 12-month time points with respect to the T/N ratio.

	6-Month Survival Rate			12-Month Survival Rate		
	OS	CSS	RFS	OS	CSS	RFS
T/N ratio						
<3	68%	68%	45%	32%	42%	11%
3–4	25%	33%	11%	17%	22%	11%
>4	67%	100%	33%	67%	100%	33%

OS, overall survival; CSS, cancer-specific survival; RFS, relapse-free survival.

## Data Availability

The data presented in this study are available from the corresponding author on reasonable request.

## Supplementary Materials

The following are available online at https://www.mdpi.com/article/10.3390/biology10040334/s1, Supplementary information: Kaplan–Meier survival curves of patients treated with BNCT only or BNCT combined with bevacizumab posttreatment. Kaplan–Meier survival curves for overall survival (**A**) cancer-specific survival (**B**) and relapse-free survival (**C**) of patients treated with BNCT only (green line) or BNCT combined with bevacizumab posttreatment (blue line). P value is marked in the upper right corner, and P values of less than 0.05 were regarded as statistically significant. OS, CSS and RFS denotes overall survival, cancer-specific survival and relapse-free survival, respectively.
