# Supplementary figures and images for: Salvage Boron Neutron Capture Therapy for Malignant Brain Tumor Patients in Compliance with Emergency and Compassionate Use: Evaluation of 34 Cases in Taiwan
Source: Biology (Basel). 2021 Apr 15;10(4):334. doi: 10.3390/biology10040334 (PMC8071294; doi:10.3390/biology10040334)

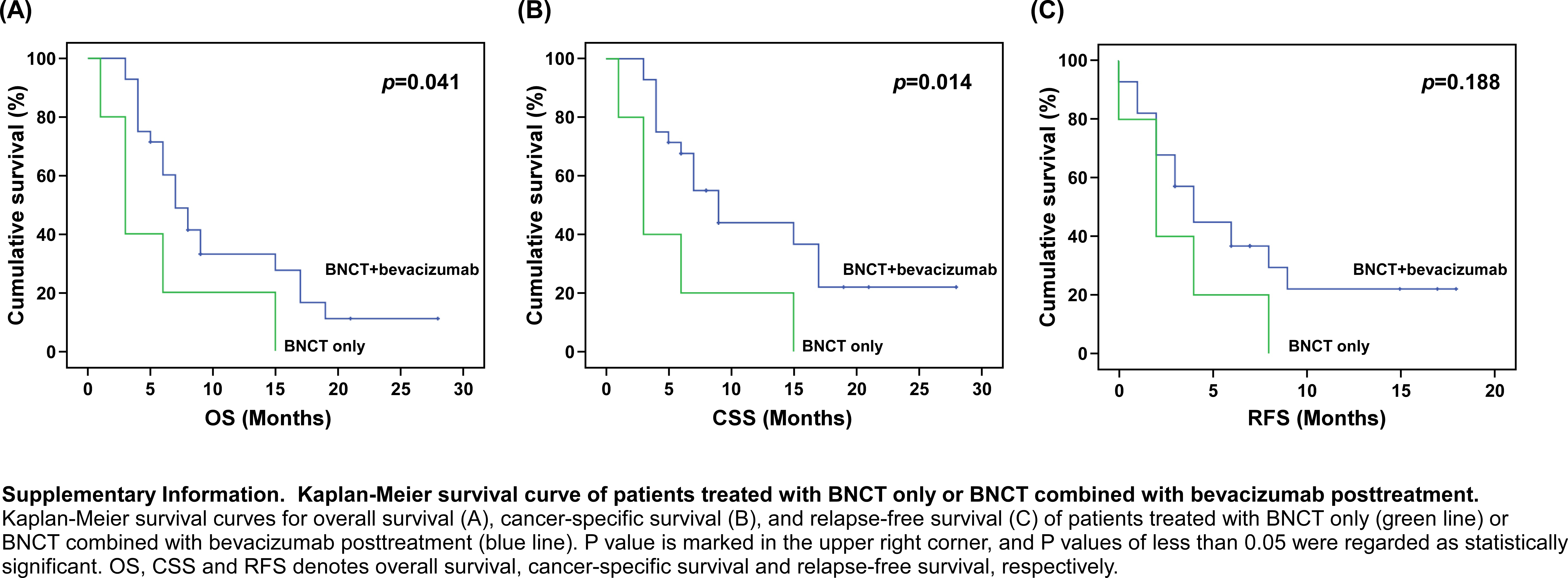

Supplement: Supplementary file 1 [file biology-10-00334-s001.zip › supplemental information.jpg]
